# Infiltration of Macrophages Correlates with Severity of Allograft Rejection and Outcome in Human Kidney Transplantation

**DOI:** 10.1371/journal.pone.0156900

**Published:** 2016-06-10

**Authors:** Tobias Bergler, Bettina Jung, Felix Bourier, Louisa Kühne, Miriam C. Banas, Petra Rümmele, Simone Wurm, Bernhard Banas

**Affiliations:** 1 Department of Nephrology, University Hospital Regensburg, Regensburg, Germany; 2 Department of Pathology, University of Regensburg, Regensburg, Germany; Institut National de la Santé et de la Recherche Médicale, FRANCE

## Abstract

**Objective:**

Despite substantial progress in recent years, graft survival beyond the first year still requires improvement. Since modern immunosuppression addresses mainly T-cell activation and proliferation, we studied macrophage infiltration into the allografts of 103 kidney transplant recipients during acute antibody and T-cell mediated rejection. Macrophage infiltration was correlated with both graft function and graft survival until month 36 after transplantation.

**Results:**

Macrophage infiltration was significantly elevated in antibody-mediated and T-cell mediated rejection, but not in kidneys with established IFTA. Treatment of rejection with steroids was less successful in patients with more prominent macrophage infiltration into the allografts. Macrophage infiltration was accompanied by increased cell proliferation as well as antigen presentation. With regard to the compartmental distribution severity of T-cell-mediated rejection was correlated to the amount of CD68^+^ cells especially in the peritubular and perivascular compartment, whereas biopsies with ABMR showed mainly peritubular CD68 infiltration. Furthermore, severity of macrophage infiltration was a valid predictor of resulting creatinine values two weeks as well as two and three years after renal transplantation as illustrated by multivariate analysis. Additionally performed ROC curve analysis showed that magnitude of macrophage infiltration (below vs. above the median) was a valid predictor for the necessity to restart dialysis. Having additionally stratified biopsies in accordance to the magnitude of macrophage infiltration, differential CD68^+^ cell infiltration was reflected by striking differences in overall graft survival.

**Conclusion:**

The differences in acute allograft rejection have not only been reflected by different magnitudes of macrophage infiltration, but also by compartment-specific infiltration pattern and subsequent impact on resulting allograft function as well as need for dialysis initiation. There is a robust relationship between macrophage infiltration, accompanying antigen-presentation and resulting allograft function.

## Introduction

The availability of calcineurin inhibitors and anti-proliferative agents as well as the introduction of costimulation blockers in recent years, which prevent activation and proliferation of T-cells, has markedly lowered acute rejection episodes. Despite these improvements in immunosuppression, acute rejection still remains a significant clinical problem, particularly with respect to the growing number of marginal organs. Since even borderline rejection is linked to impairment of graft function and premature graft loss [1–3], acute rejection represents an ongoing immunological risk factor, e.g. for subsequent interstitial fibrosis and tubular atrophy (IFTA)[4].

The pivotal role of T-lymphocytes in the initiation of acute rejection has generally been accepted. However, there are inconsistencies regarding the role of other cell types such as macrophages: on the one hand, it has been recognized that sole T-cell infiltration and even tubulitis is not necessarily linked to impaired graft function [5;6]. On the other hand, due to the observation that some patients even develop acute cellular rejection after T-cell depleting induction therapy, it has been recognized that T-cells cannot be the only infiltrating cell population initiating graft rejection.

Macrophages, as key elements of innate immunity, are present within transplanted kidneys contributing to acute and chronic allograft injury by a variety of mechanisms [7]. Because of their predominating presence during acute rejection episodes, macrophages have initially been thought to be contributors to T-cell-mediated graft injury [8]. With increasing knowledge of macrophage biology, a wider range of macrophage functions has become evident, including the modulation of inflammation, the participation in innate as well as adaptive immunity, and the contribution to tissue injury and repair [8;9].

In organ transplantation, accumulation of macrophages was verified in models of acute as well as chronic injury. In biopsies of acute allograft rejection macrophages can account for up to 60% of infiltrating leucocytes, accumulating in different renal compartments, e.g. interstitial, perivascular and glomerular [10]. However, the presence of macrophages in donor organs decreases gradually, beginning at an early stage after transplantation [11].

Since current baseline immunosuppression focusses mainly on prevention of T-cell activation and proliferation, we were interested to better define the role of macrophages in kidney transplantation. First, we were interested in the extent of macrophage infiltration in subtypes of renal allograft rejection (antibody mediated rejection [ABMR]; T-cell mediated rejection [TCMR] without and with arteritis) in comparison with normal histology and chronic alteration (interstitial fibrosis/tubular atrophy [IFTA]). Secondly, we analysed macrophage infiltration into different renal compartments (peritubular, glomerular, perivascular) according to histopathological diagnosis. In a third step we analysed outcome data of different rejection categories and correlated the severity of macrophage infiltration with creatinine values up to 36 months post-transplantation as well as with overall graft survival in an observation for more than ten years after renal transplantation. In addition to sole macrophages infiltration into the graft, we looked for properties of cell proliferation and antigen presentation expressed by infiltrating macrophages.

## Methods

### Patients/human renal allograft biopsies

In our transplant center, protocol biopsies are routinely performed 2 weeks and 3 months after transplantation. Additional indication biopsies at earlier time points after renal transplantation typically were performed due to allograft dysfunction, e.g. stagnating/inadequate falling creatinine. At later time points indication biopsies usually were performed to exclude acute or chronic rejection, drug toxicity or recurrence of the primary kidney disease, resp. Since 1998 all recipients of a kidney transplantation in our transplant center, except a small number of patients, who refused consent, were observed for their clinical course after transplantation. Clinical characteristics before and after transplantation, features of transplantation (i.e. donor data), course of graft function, complications etc. as well as histologic and diagnostic results were collected in a big database. Here from a total of 103 randomly chosen patients (transplanted between 2000 and 2007) and 103 corresponding formalin-fixed, paraffin-embedded renal biopsies were included in the analysis (patient characteristics are given in Table 1). Each biopsy was graded according to the Banff ‘09 classification [12] by a single pathologist. We randomly included patients with normal histology (no rejection/no IFTA), with antibody-mediated rejection (ABMR), with T-cell mediated rejection (TCMR) of different severity and patients with already established chronic renal damage (IFTA). To avoid confounding effects we excluded cases with combined appearance, such as mixed ABMR/TCMR or acute rejection with accompanying IFTA. All biopsies with antibody-mediated rejection were C4d positive, but only 1 of 46 biopsies (2.2%) with active TCMR.

**Table 1:** Patient characteristics of entire study population.

**number of patients (n)**	**103**
**kind of transplantation (n; percent)**	** **
postmortal	**42 (41)**
living donation	**20 (20)**
ET senior	**24 (23)**
rescue offer	**2 (2)**
2nd/3rd transplantation	**8 (8)**
combined kidney pancreas transplantation	**7 (7)**
**donor age [median; interquartile range]**	**52 [22–80]**
**donor sex (male/female)**	**56/47**
**recipient age [median; interquartile range]**	**57 [16–75]**
**recipient sex (male/female)**	**73/30**
**cold ischemia time in min**	**841 [60–2280]**
**HLA-mismatch**	**3 [0–6]**
**PRA current [median; interquartile range]**	**[26; 11–62]**
**PRA highest [median; interquartile range]**	**[36; 7–100]**
**Pts. with donor specific antibodies post-tx (n)**	**10**
**delayed graft function (n; percent)**	**19 (18)**
**UPCR in g/g 12 month postTX (72 patients)**	**0.12 [0.03–1.55]**
**UPCR in g/g 36 month postTX (70 patients)**	**0.13 [0.02–3.56]**
**creatinine in mg/dl (median; range)**	** **
12 month post TX (89 patients)	**1.95 [0.84–4.53]**
36 months post TX (79 patients)	**1.84 [0.87–3.58]**
**indication biopsy (n;percent)**	**60 (58.3)**
**Overall graft survival in months (median)**	**87 **
graft survival after 3 years (n; percent) (4 pts. with primary non-function excluded)	78/99 (79%)
graft survival after 5 years (n; percent) (4 pts. with primary non-function excluded)	72/99 (73%)

Illustrated patient characteristics independent of underlying histopathological classification (UPCR = urine protein: creatinine ratio

All renal transplant recipients were treated with triple immunosuppressive therapy including prednisolone, which was administered to all patients for at least 3 months after transplantation, tacrolimus or cyclosporine A and mycophenolate mofetil or mycophenolic acid (see Table 2, last row). No patient received T-/B-cell depleting induction therapy. Human tissue was analysed according to the valid approval of the Ethics Committee of the Medical Faculty of the University of Regensburg and written informed consent from the donors of the samples of the biological material was obtained—before renal transplantation. Rejection episodes were treated with steroid-pulse for at least 3 days (250 mg/day) and/or ATG Fresenius®/thymoglobulin in cases of severe, ongoing TCMR until biopsies showed complete disappearance of rejection. ABMR patients were treated with steroids+thymoglobulin and/or plasmapheresis/IVIG.

**Table 2:** Baseline characteristics of human allograft biopsies according to the histopathological grading.

	normal histology	ABMR	TCMR-	TCMR+	IFTA
**number of patients (n)**	34	7	14	32	16
**recipient sex** (male/female)	25/9	3/4	12/2	28/4	13/3
**recipient age in years** (mean, SD)	54.1 ± 13.5	57.1 ± 14.7	55.5 ± 13.6	56.4 ± 12.3	50.5 ± 16
**organ donation** (cadaver/living)(n)	23/11	5/2	10/4	22/10	12/4
**donor sex** (male/female)(n)	16/18	3/4	9/5	15/18	11/5
**donor age in years** (mean, SD)	51.4 ± 13.7	56.4 ± 10.7	51.5 ± 16.4	54.2 ± 12.6	62 ± 14.6
**MHC mismatches** (mean, SD)	3.2 ± 1.7	**4.5 ± 1.1**^#^	3.6 ± 1.7	3.8 ± 1.5	3.5 ± 1.5
**PRA highest (%)** (mean,SD)	3.1 ± 11.4	**17.4 ± 25.5**^#^	5.6 ± 16.2	5.7 ± 14.5	3.4 ± 10.0
**cold ischemia time** (min) (mean, SD)	652 ± 489	597 ± 422	549 ± 324	720 ± 451	633 ± 181
**delayed graft function** (n, percent)	4 (12%)	1 (14%)	2 (14%)	**12 (38%)**^#^	3 (19%)
**time point of biopsy postTX** (days) (mean, SD)	31.0 ± 31.7	19.4 ± 4.4	27 ± 29.2	17.4 ± 18.2	**2017 ± 486**^#^
**creatinine at biopsy** (mg/dl)(mean, SD)	2.7 ± 1.9	2.6 ± 0.9	3.1 ± 1.4	**4.7 ± 3.1**^#^	3.1 ± 2.0
**indication biopsy** number (percent)	4 (11%)	5 (71%)	10 (71%)	27 (84%)	14 (88%)
**immunosuppression (IS)** § triple IS (percent)/percentage of tacrolimus based IS	97%/94%	100%/100%	93%/93%	97%/87.5%	37.5%/81%

Significant differences in comparison with normal histology (p < 0.05) are highlighted.

^#^ illustrates statistical significance.

^§^ regime of immunosuppression was continuously reported.

ABMR: antibody mediated rejection; TCMR-: tubulointerstitiell rejection without arteritis; TCMR+: tubulointerstitiell rejection with arteritis; IFTA: interstitiell fibrosis/tubular atrophy

#### Immunohistochemistry

Immunohistochemistry was performed on 3μm sections as described previously [13]. As primary antibodies, a monoclonal mouse anti-human CD68 (1:100, M0814,clone KP-1, DAKO, Hamburg, Germany), a polyclonal rabbit anti-human Ki67 (1:50, ab833, abcam, Cambridge, UK), and a monoclonal rat anti-human MHCII-HLA-DR antibody (1:50, MA1-70112 clone YE2/36 HLK, ThermoScientific, Rockford, IL, USA) were used.

#### Analysis of cellular infiltration

For Histoquest^**®**^ software-based analysis, digital pictures were taken and 10 high power fields (HPF) per specimen were examined for analysis (original x 400, covering an area of 296 μm x 222 μm) of each biopsy as previously described [14]. Only areas in the cortex were analysed. With the Histoquest^®^ software the number of CD68, Ki67, and MHCII-HLA-DR-positive cells (identified by a double staining of DAPI+specific antibody) related to all cells (only DAPI-positive) and related to the defined area were automatically counted.

The slides were evaluated by light microscopy. The positive CD68, Ki67, and MHCII-HLA-DR staining in the peritubular, the glomeruli, and the perivascular compartment was analysed by two investigators in the whole tissue of human biopsies. It was analysed in at least 10 HPF of 2 different areas (5 neighbouring HPF in each area) within the cortex as previously described [14]. Mean values were calculated and used for comparison of the different histological categories according to Banff classification.

#### Double-immunofluorescence

Double-labelling immunofluorescence was performed on paraffin-embedded sections as described previously [13]. In human biopsies, CD68 double staining was performed using Ki67 (1:50, ab833, abcam, Cambridge, UK), or MHCII-HLA-DR (1:50, MA1-70112 clone YE2/36 HLK, ThermoScientific, Rockford, IL USA). Primary antibodies were detected by AlexaFluor antibodies (Invitrogen, Karlsruhe, Germany) or by biotin (DAKO, Hamburg, Germany) and HRP (Dianova, Hamburg, Germany). Quantification of CD68-MHCII-HLA-DR and CD68-Ki67 costaining was done in a random sample of biopsies with TCMR (n = 15 biopsies).

#### Statistical analysis

Receiver-operating characteristics (ROC) analyses were conducted to differentiate between patient groups (below/above the median of CD68 counts) and need for dialysis 12 and 36 months after renal transplantation. Estimates for the area under the curve (AUC) with the corresponding 95% confidence interval (CI) were reported. All analyses were done with IBM SPSS Statistics 20.0.0.

## Results

### Patient and histological characterization

Overall graft survival was 87 months [1–170]. In the overall patient population with graft failure need for dialysis occurred in median after 45 months [1–170]. Patient characteristics according to the histopathological grading are presented in Table 2. Biopsies with ABMR had a significantly higher number of MHC mismatches (p = 0.01) and a significantly higher number of panel reactive antibodies (PRA) (p = 0.009). Biopsies with TCMR with arteritis (TCMR+) were significantly more often affected by delayed graft function (DGF) (p = 0.004) and displayed significantly elevated creatinine values at biopsy (p = 0.004).

Having analysed acute rejecting patients below and above the median of CD68 counts we found that in the below from the median group 20/44 patients (45.45%) had no rejection, but only 14/43 patients (32.56%) in the above from the median group. 3/44 patients (12.5%) in the below from the median group had to be treated with additional ATG, due to steroid-resistant rejection, but 18/29 patients (61.1%) in the above from the median group.

Focussing on the clinical outcome, biopsies with TCMR with arteritis had a significantly increased CD68 infiltration, which was reflected by significantly elevated creatinine values up to month 36 after transplantation, but no differences regarding graft survival in comparison with normal histology (Table 3). In contrast biopsies with ABMR illustrated not only a significantly increased CD68 infiltration, but also an elevated creatinine value 12 months after transplantation (p < 0.0001), a significantly increased proteinuria (p = 0.01) and a significantly shortened graft survival (p = 0.008) (Table 3).

**Table 3:** Clinical outcome data.

	normal histology	ABMR	TCMR-	TCMR+	IFTA
**CD68 count**	84 ± 138	**338 ± 555**^#^	**258 ± 344**^#^	**451 ± 518**^#^	279 ± 666
**creatinine at biopsy** (mg/dl)	2.7 ± 1.9	2.6 ± 0.9	3.1 ± 1.4	**4.7 ± 3.1**^#^	3.1 ± 2.0
**creatinine 2 weeks postTx** (mg/dl)	2.6 ± 1.6	3.7 ± 1.3	3.1 ± 1.7	**4.5 ± 3.4**^#^	3.7 ± 2.3
**creatinine 12 months postTX** (mg/dl)	1.7 ± 0.5	3.1 ± 1.1^#^	1.9 ± 0.5	**2.4 ± 1.6**^#^	2.4 ± 0.8
**creatinine 36 months postTX** (mg/dl)	1.7 ± 0.6	1.9 ± 0.2	2.0 ± 0.6	**2.8 ± 0.8**^#^	2.0 ± 0.8
**proteinuria 12 months postTx** (mg/g crea)	148 ± 136	**450 ± 734**^#^	93 ± 43	201 ± 200	115 ± 105
**proteinuria** (mg/g crea-36mths)	324 ± 456	643 ± 660	247 ± 355	527 ± 1029	484 ± 840
**graft survival** (months)	81 ± 35	**46 ± 33**^#^	91 ± 30	69 ± 43	74 ± 52

**Outcome data** of different patient populations according to the underlying histopathological grading are shown. Significant differences in comparison with normal histology (p < 0.05) are highlighted.

^#^ illustrates statistical significance.

ABMR: antibody mediated rejection; TCMR-: tubulointerstitiell rejection without arteritis; TCMR+: tubulointerstitiell rejection with arteritis; IFTA: interstitiell fibrosis/tubular atrophy

#### Software-based analysis of macrophage infiltration, antigen presentation and proliferation

**Fig 1:**
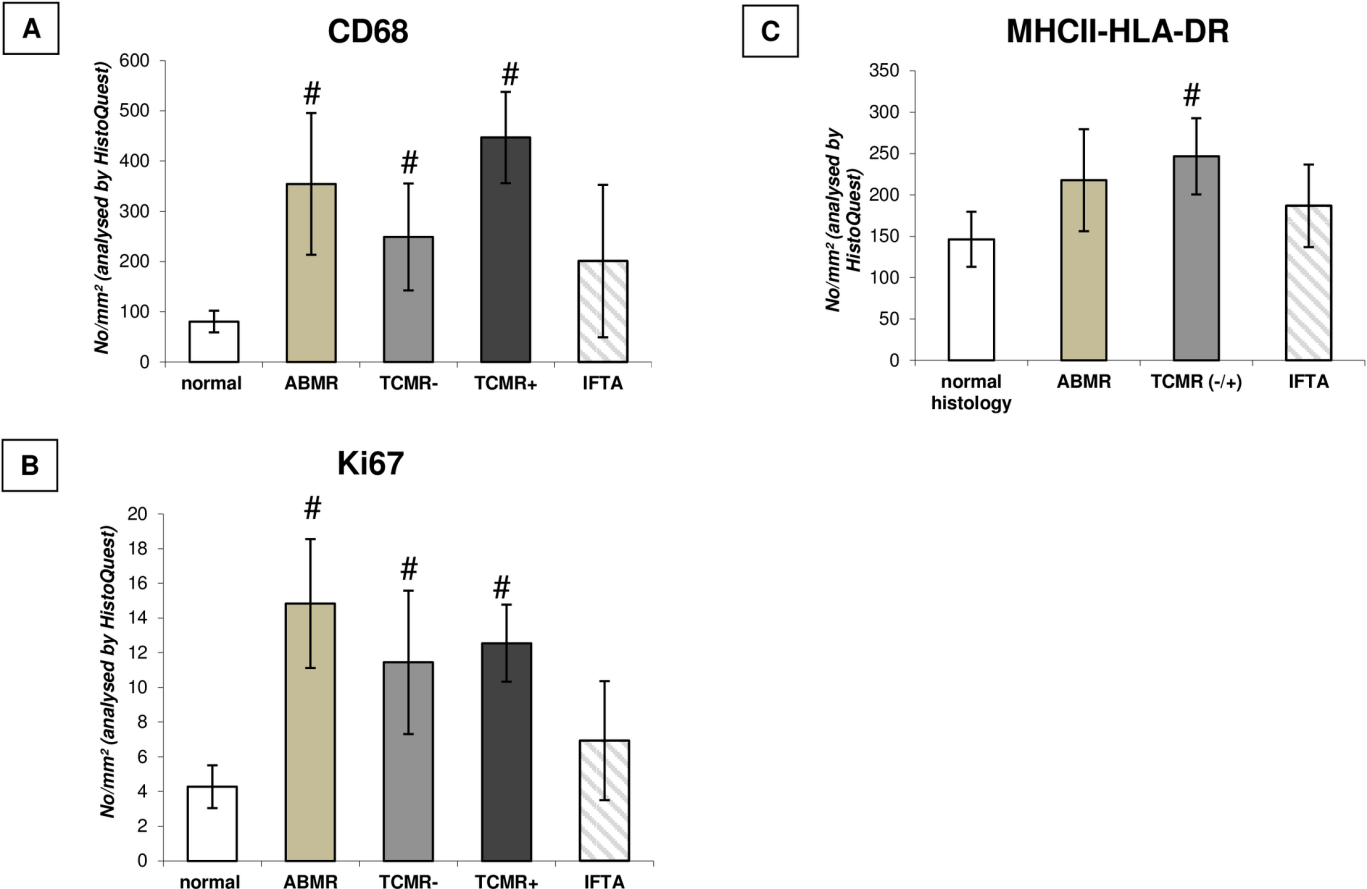
Allograft rejection enhances cellular infiltration. Quantity of CD68 (A), Ki67 (B), and MHCII-HLA-DR (C) positive cells in renal allograft biopsies dependent on underlying histopathological category. # p < 0.05 vs. normal histology. ABMR = antibody mediated rejection, TCMR- = T-cell mediated rejection without arteritis; TCMR+ = T-cell mediated rejection with arteritis; IFTA = interstitial fibrosis/tubular atrophy.

Quantification of CD68 infiltration, Ki67 expression, and MHCII-HLA-DR-dependent antigen presentation was performed in biopsies according to the histopathological diagnosis by Histoquest^®^ as shown in Fig 1A–1C. We detected a significantly increased infiltration with CD68+ cells, Ki67+cells and MHCII-HLA-DR+ cells in acute rejecting grafts (ABMR/TCMR), whereas grafts with interstitial fibrosis/tubular atrophy (IFTA) showed no modified expression of these markers. There was no significant difference in macrophage infiltration between grafts with ABMR and TCMR for the overall analysis.

In a random sample of 15 biopsies with TCMR without and with arteritis we found that 79.1% ± 2.3 of MHCII-HLA-DR+ cells were also CD68+ positive, whereas 39.3% ± 8.7 of Ki67+ cells were also CD68+.

#### Compartment-specific analysis of macrophage infiltration and proliferation

**Fig 2:**
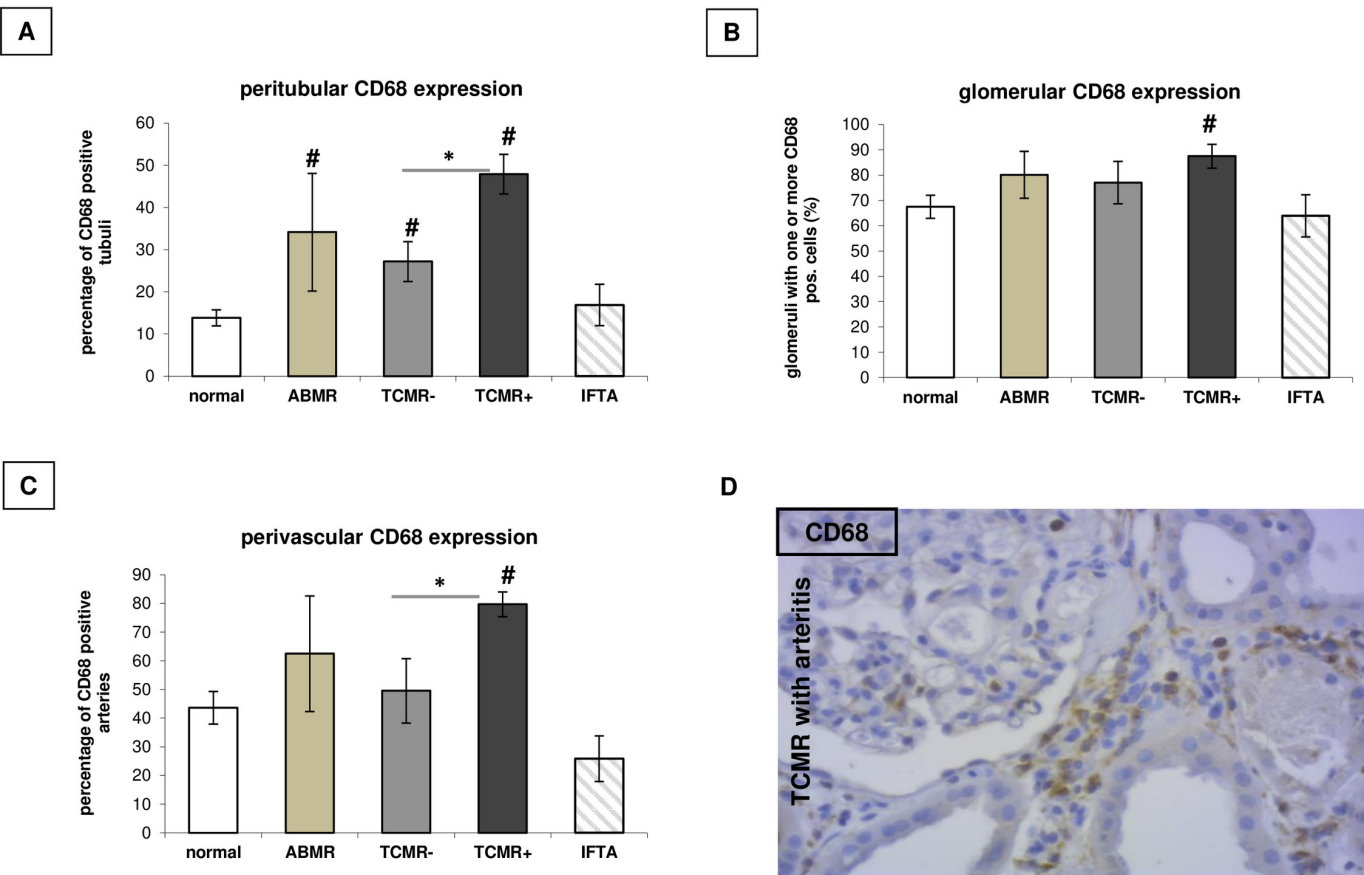
Compartment-specific macrophages expression. Compartment-specific expression (peritubular (A), glomerular (B), perivascular (C)) of **CD68 positive macrophages** in renal allograft biopsies dependent on histological grading. # p < 0.05 vs. normal histology; * p < 0.05 vs TCMR without arteritis. D: CD68 expression in a representative biopsy (TCMR with arteritis).

**Fig 3:**
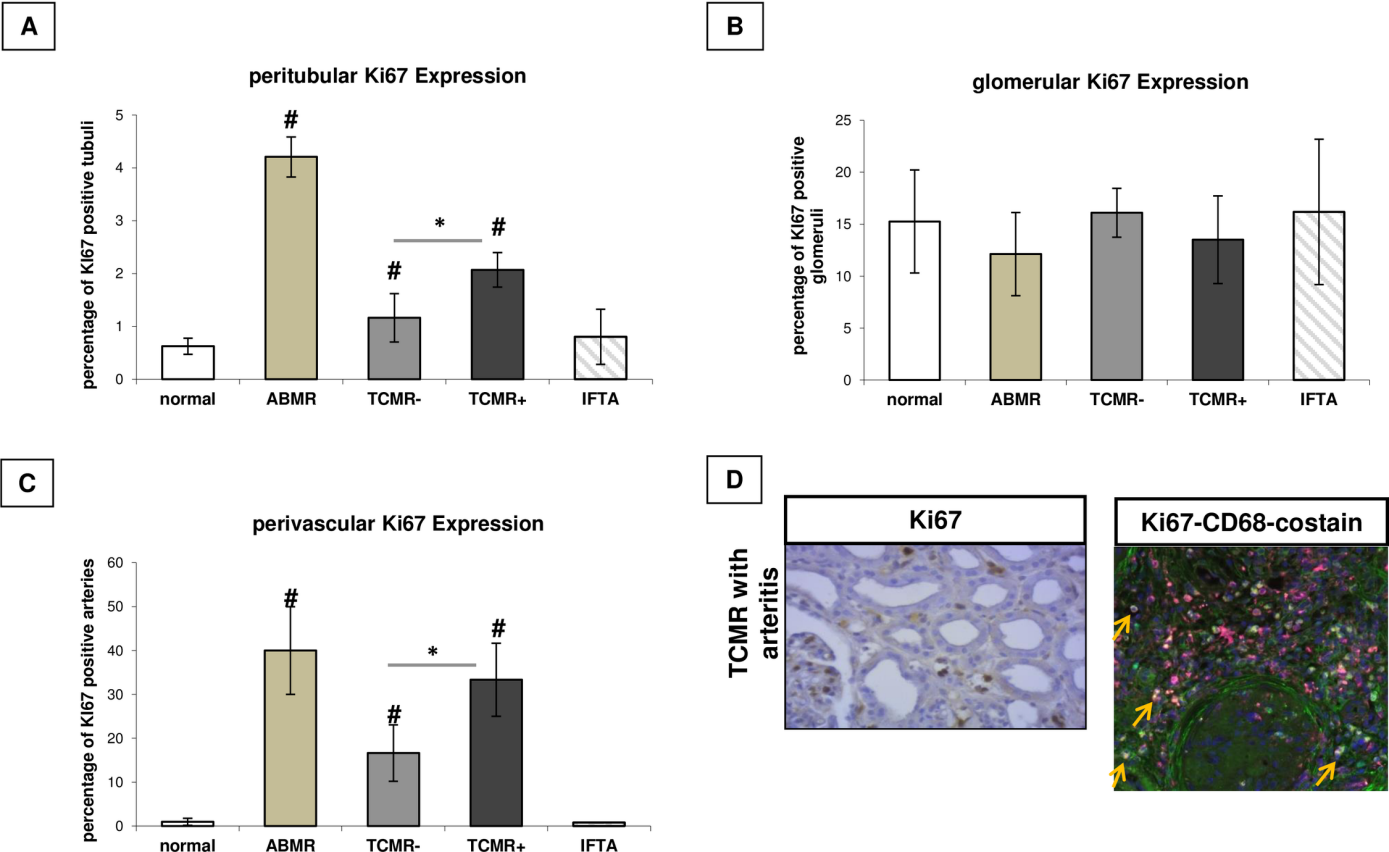
Compartment-specific cellular proliferation. Compartment-specific expression (peritubular (A), glomerular (B), perivascular (C)) of **Ki67** positive cells in renal allograft biopsies dependent on histopathological grading. # p < 0.05 vs. normal histology;; * p < 0.05 vs TCMR without arteritis. D: left-hand side: Ki67 expression in a representative biopsy (TCMR with arteritis); right-hand side: representative example of a Ki67 (green) /CD68 (red) costaining (orange arrow).

**Fig 4:**
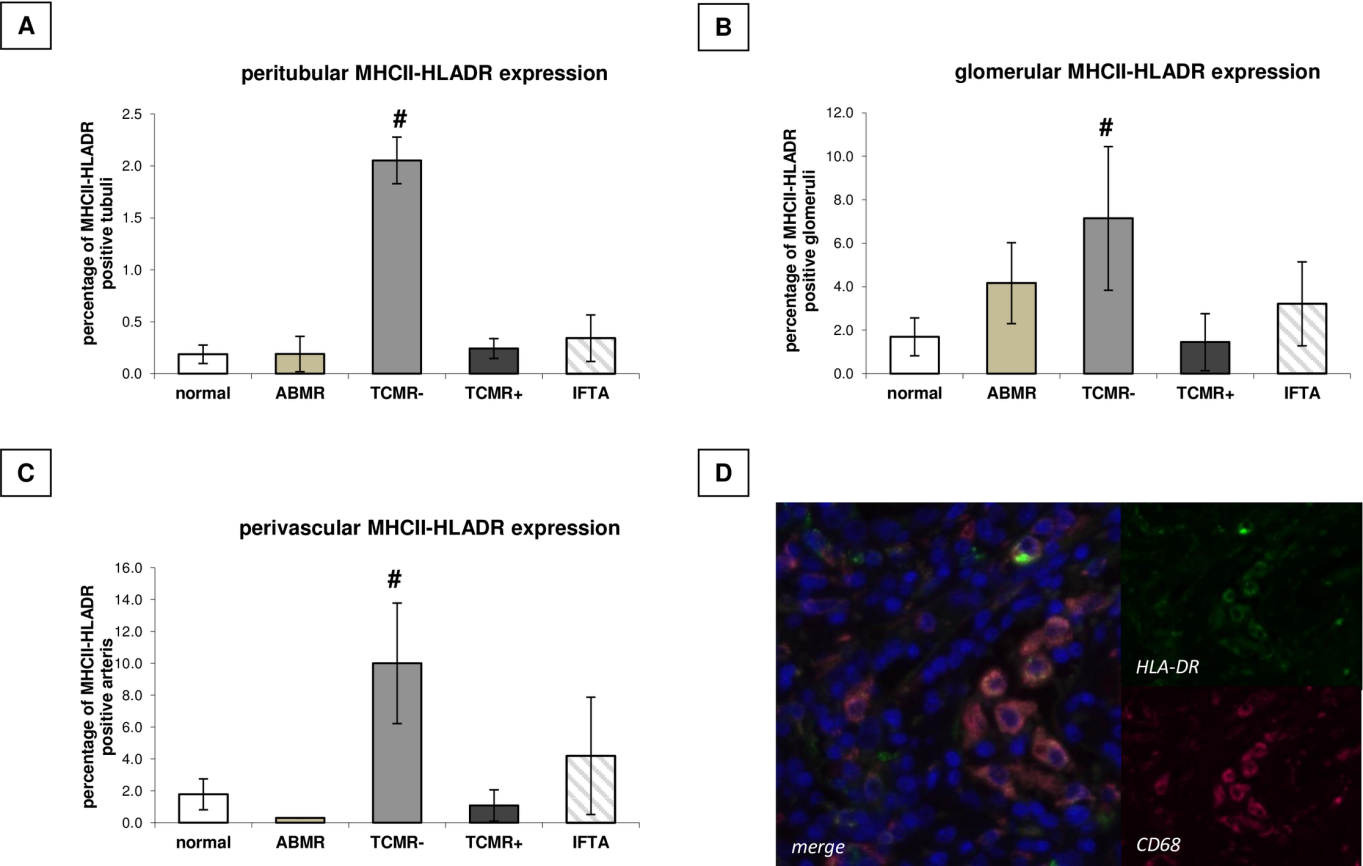
Compartment-specific antigen expression. Compartment-specific expression (peritubular (A), glomerular (B), perivascular(C)) of **MHCII-HLA-DR** positive cells in renal allograft biopsies dependent on histopathological grading. # p < 0.05 vs. normal histology. D: Representative slide of a CD68-MHCII-HLADR costaining in a TCMR with arteritis biopsy.

Infiltration of CD68^+^, Ki67^+^ and MHCII-HLA-DR^+^ cells was analysed manually in the peritubular, glomerular, and perivascular compartment of the different renal biopsies. On average, 27.2 tubuli, 5.3 glomerula, and 5.3 arteries were analysed per biopsy, in which the median value for tubules/biopsy was 55, 9.2 for glomerula and 10 for arteries. Severity of TCMR is transferred to the amount of CD68 infiltration in the examined renal compartments (Fig 2A–2C). Additionally in the peritubular (p = 0.03) as well as the perivascular compartment (p = 0.004) aggressive TCMR with accompanying arteritis showed a significantly increased CD68 infiltration in contrast to TCMR without arteritis. Antibody-mediated rejection showed only a significantly increased CD68 infiltration in the peritubular compartment (Fig 2A), with a trend for an increased infiltration to the glomerula (Fig 2B). But having accounted only for biopsies with more than 3 CD68 positive cells within the glomeruli, only patients with ABMR retained positive. Ki67 also correlated with the severity of TCMR, but also with antibody-mediated rejection in the peritubular and the perivascular compartment (Fig 3A and 3C), without any significant differences in the glomerular compartment (Fig 3B). MHCII-HLA-DR expression was only induced in the context of mild TCMR without arteritis (Fig 4A–4C).

#### CD68+ infiltration and graft outcome

*First* we compared the amount of macrophage infiltration in accordance to the underlying histological grading (normal histology, antibody-mediated rejection and T-cell-mediated rejection with and without arteritis, IFTA) and features of graft outcome. We found that increased macrophage infiltration—especially during TCMR with arteritis—was associated with impaired renal function up to month 36 after transplantation. However, graft survival was only limited in ABMR biopsies (p = 0.008) being accompanied by increased proteinuria 12 months after renal transplantation (Table 3).

**Fig 5:**
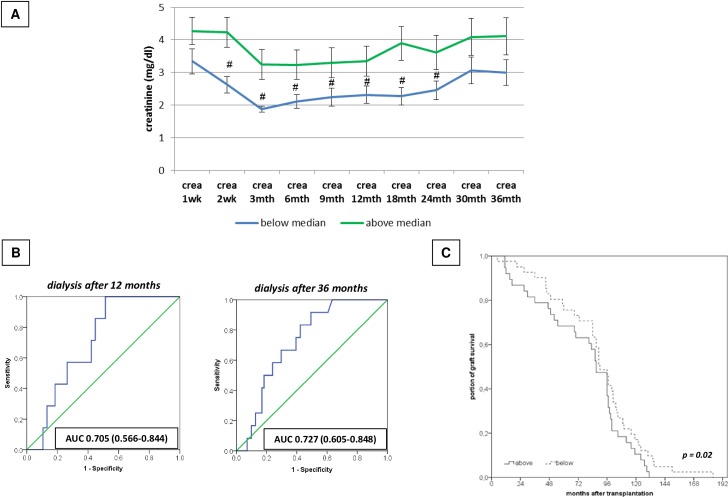
Consequences of macrophages infiltration for renal function and graft survival. A: Association of CD68+ cell infiltration (below vs. above the median of macrophage infiltration) with corresponding serum creatinine values until 36 months after transplantation. # p < 0.05. B: ROC curve analysis on impact of CD68 infiltration for need for dialysis initiation (12months/36 months after transplantation). C: Kaplan-Meier curve for corresponding overall graft survival (below vs. above the median)

*Secondly* we addressed the question, if macrophage infiltration per se—*independent of the underlying histological grading*—is associated with impaired renal function. Having identified the magnitude of macrophage infiltration in the total cohort of biopsies (n = 103) (range: 0–2245 CD68 counts), we then opposed biopsies below the median of macrophage infiltration [2–83] (n = 51) to biopsies above the median of macrophage infiltration [100–2245] (n = 52). The mean value of CD68 counts in biopsies below the median was 28 ± 21. In contrast, the mean value of CD68 counts in biopsies above the median was 513 ± 538 counts (p = 5.2E-8). Comparing corresponding creatinine values until 36 months after transplantation showed that averaged creatinine values of the biopsies below the median of CD68^+^ infiltration (2.5 ± 1.9 mg/dl) were significantly lower than averaged creatinine values of the biopsies above the median of CD68^+^ infiltration (3.7 ± 3.2 mg/d; p = 1.2E-5) (Fig 5A). Additionally need for dialysis in the below from the median group occurred after in median time 59 months and in the above from the median group after 22.5 months.

Multivariate analyses (MANOVA) on magnitude of macrophages infiltration and other well known risk factors for longterm allograft function (donor age, donor weight, cold ischemia time (CIT), number of MHC mismatches [HLA-A, HLA-B, HLA-DR], delayed graft function (DGF), recurrence of primary kidney disease, peak-PRA) showed, that magnitude of macrophages infiltration had a significant impact on resulting creatinine values 2 weeks (p = 0.003), 2 years (p = 0.018) and three years (p = 0.014) after renal transplantation. Besides magnitude of macrophages infiltration only DGF had a significant *between subject effect* (p = 0.044) for resulting creatinine values two week, two years and three years after transplantation. For the length of CIT only a trend (p = 0.063) was seen.

To account for a clinical endpoint of above stated CD68 counts (below vs. above the median of CD68 infiltration) we *thirdly* performed ROC curve analysis for the resulting need for dialysis 12 and 36 months after transplantation: ROC curve analysis illustrated that there is a robust influence of the degree of CD68 infiltration (below vs above the median) for dialysis initiation 12 months (AUC 0.705; CI 0.566–0.844) as well as 36 months after transplantation (AUC 0.727; CI 0.605–0.848) (Fig 5B). Multivariate analysis additionally showed a significant impact of the magnitude of macrophages infiltration on need for dialysis after 36 months (p = 0.016). Creatinine values after 2 years were only a weak predictor for dialysis initiation 36 months after transplantation (AUC 0.633; CI 0.430–0.835).

Additionally we *fourthly*, accounted for overall graft survival (until year 14 postTx) in biopsies below and above the median of macrophages infiltration: biopsies below the median had a significantly better graft survival than biopsies above the median of macrophages infiltration: 89 ± 36 months (median 90 [5–184]) vs 68 ± 42 months median 86 [3–131], p = 0.02) (Fig 5C).

## Discussion

In contrast to the role of T-cells, knowledge on macrophages in allograft rejection is still lacking [15]. It is generally accepted that macrophage infiltration is present in the early phase of transplantation due to ischemia-reperfusion injury and is associated with graft dysfunction as well as shortened graft survival [16;17]. Newer publications, focussing on macrophages subpopulations presumed impact for IFTA [18] and benefits of monocytes monitoring [19]. However, there are some inconsistencies regarding the role of macrophages in different types of allograft rejection and chronic allograft damage, and only very few studies have investigated the effects of macrophages infiltration for longterm graft survival.

In the present study of renal transplant biopsies the number of infiltrating macrophages correlated with the severity of acute rejection, as well as with renal function and long-term allograft function. Multivariate analysis clearly demonstrated, that besides severity of macrophages infiltration only delayed graft function (DGF) had a significant impact, in contrast to several other well-known factors for long-term renal outcome, (e.g. number of HLA-mismatches, duration of CIT, recurrence of primary kidney disease). The importance of macrophages was independently underlined by the finding, that more severe macrophages infiltration was associated with higher rates of dialysis re-initiation 12 and 36 months after transplantation.

Compartment-specific infiltration of CD68^+^ cells especially into the peritubular and the perivascular compartment was linked with TCMR with arteritis. Furthermore, costaining experiments illustrated that infiltrating macrophages also displayed induced HLA-DR antigen presentation.

The presented data of macrophage infiltration in antibody-mediated rejection as well as in T-cell-mediated rejection from human renal transplant biopsies expands existing data on the role of macrophages as initiators of graft injury and predictors of graft outcome [20–24]. This is strongly underlined by our finding that the severity of macrophage infiltration—being mostly increased during TCMR with arteritis—is accompanied by impaired creatinine values up to month 36 post transplantation. Costaining experiments illustrated that the majority of HLA-DR+ cells is CD68 positive (80%), so that a significant impact of other cell subtypes, e.g. B-lymphocytes as HLA-DR source can be disregarded. The detected differences in macrophages infiltration were independent of the length of cold ischemia time, but dependent on the accompanying delayed graft function (DGF), also verified by MANOVA analysis. Thus, increased CD68 infiltration with accompanying HLA-DR expression—binding and presenting peptides from antigens on the cell surface for recognition by T-cells—links innate and adaptive immune system. However, uptodate no pathomechanistical link between higher CD68 cell infiltration/HLA-DR expression and graft survival can be offered, but the CD68+HLA-DR+ mononuclear cells may be regarded as a predictive marker for longterm graft survival.

Findings on the severity of macrophage infiltration—also verified by compartmental specific examinations—being associated with the severity of TCMR are in line with previous investigations [10;25–27]. However, we have not only confirmed these data, but added that the severity of TCMR is also reflected by the peritubular CD68 expression. Additionally, severe TCMR with accompanying arteritis was linked with glomerular and perivascular macrophage infiltration, an observation that may even be related to the later development of allograft glomerulopathy [28;29]. Messias et al., also postulated, that glomerulitis was associated with vascular rejection [30], however we found this not only for highly sensitized patients, as our TCMR+ patients had no higher levels of PRA or HLA-mismatches in comparison with patients with normal histology.

Glomerular CD68 expression is regarded as a surrogate for antibody-mediated rejection, where we found only a trend for an increased CD68 infiltration. However, the detected results remain somehow contradictory: if one accounts for any CD68 positive cell within the glomeruli even biopsies with normal histology exhibit up to 70% of CD68 positive glomeruli. However, if one focusses only on glomerula with more than 3 CD68+ cells, only patients with ABMR retained positive. Additionally, to ensure the correctness of histopathological classification we analysed C4d staining of examined biopsies, which were only positive in the case of antibody-mediated rejection. Therefore combined appearances (ABMR+TCMR) as source of glomerular CD68 infiltration can be probably neglected, however, cases of C4d negative antibody-mediated rejection can´t be ruled out [31]. Discrepancies in detected glomerular macrophage infiltration in our observations in comparison with previous reports may somehow also be explained by the availability of more potent immunosuppression today, the exclusion of nephrectomy specimens, glomerulonephritis [32;33] or CMV viremia [34] and the identification of monocytes using specific antibody staining rather than electron microscopy.

In the current study, entire as well as compartment specific macrophage infiltration is linked to the severity of TCMR and resulting kidney function. The significance of the present results was broadened by the additional finding that macrophage infiltration was accompanied by compartment specific cell proliferation–illustrated by Ki67 expression. This may indicate that infiltrating macrophages act as an ongoing trigger of subclinical alloimmune inflammation and subsequent progression of graft injury. Recently published data by Toki et al. on the role of macrophages for the development of renal fibrosis support this hypothesis [35]. Our data, that acute rejection is characterized by infiltrating as well as proliferating macrophages, confirm data published by Seron et al., who characterized renal biopsies for expression of different proliferation markers, e.g. Ki67 [36].

In contrast to acute rejecting grafts biopsies from grafts with chronic damage and established interstitial fibrosis and tubular atrophy showed no induced macrophages infiltration, cell proliferation or antigen presentation. While experimental and human studies have highlighted the impact of macrophages for the development of IFTA [37–41], the impact of macrophages in already established IFTA seems very limited.

In conclusion, using both software-based as well as compartment-specific analyses, we can clearly demonstrate that CD68 infiltration is strongly linked to acute antibody-mediated and T-cell-mediated allograft rejection. Severe TCMR with accompanying arteritis significantly affected resulting creatinine values up to 36 months after transplantation. Additional consideration of the magnitude of macrophages infiltration (below vs. above the median of CD68 infiltration) irrespective of the underlying diagnosis independently affected resulting renal function: the lower the magnitude of macrophages infiltration the better the resulting creatinine values. A result being verified by ROC curve analysis as well as corresponding graft survival more than 10 years after transplantation. The AUC for the predictive value of macrophages infiltration for emerging need for renal replacement therapy 12 and 36 months after renal transplantation were as robust as were the differences for graft survival in dependence to the magnitude of macrophage infiltration.

Thus, these findings emphasize, that macrophages influx into renal allografts is an important risk factor for deterioration of renal function and a predictive indicator for rejection outcome.
